# Implementation and Scale-Up of Psycho-Trauma Centers in a Post-Conflict Area: A Case Study of a Private–Public Partnership in Northern Uganda

**DOI:** 10.1371/journal.pmed.1001427

**Published:** 2013-04-16

**Authors:** Etheldreda Nakimuli-Mpungu, Stephen Alderman, Eugene Kinyanda, Kathleen Allden, Theresa S. Betancourt, Jeffrey S. Alderman, Alison Pavia, James Okello, Juliet Nakku, Alex Adaku, Seggane Musisi

**Affiliations:** 1 Makerere University, College of Health Sciences, Department of Psychiatry, Kampala, Uganda; 2 Butabika National Referral Mental Hospital, Kampala, Uganda; 3 Peter C. Alderman Foundation, Bedford, New York, United States of America; 4 Medical Research Council, Entebbe, Uganda; 5 Dartmouth University, Geisel School of Medicine, Department of Psychiatry, New Hampshire, United States of America; 6 Harvard School of Public Health, Boston, Massachusetts, United States of America; 7 Gulu University, Department of Psychiatry, Gulu, Uganda; 8 Arua Regional Referral Hospitals, Arua, Uganda

## Abstract

As one article in an ongoing series on Global Mental Health Practice, Etheldreda Nakimuli-Mpungu and colleagues describe a private-public partnership that implemented and scaled psycho-trauma centers in Northern Uganda.

**Summary Points**

- The Peter C. Alderman Foundation (PCAF) and Ugandan government institutions initiated a public–private partnership (PPP) demonstrating the feasibility of delivering low cost, evidence-based mental health care to massively traumatized populations in northern Uganda.
- The PPP employed a systems approach to mental health care, wherein clinics could deliver uniform treatment that was locally adapted to each tribal culture.
- The PPP leveraged its pooled resources, raising the value of patient care to a level that none of the partners could provide by working alone.
- The PPP established metrics to assess the impact of therapy on war-affected people remaining in their own country after the cessation of hostilities. The ongoing prospective evaluation of PCAF program participants offers valuable information on the potential benefits of treating depression, post-traumatic stress disorder, and other mental, neurological, and substance use disorders in post-conflict low- and middle-income countries.

*This case study is part of the* PLOS Medicine *series on Global Mental Health Practice.*

## Introduction

Since declaring independence from the British in 1962, the Republic of Uganda has experienced three-and-a-half decades of protracted civil war. No region of the country has been spared. In 1987, the Holy Spirit Uprising [1] gave rise to renewed conflict in the northern regions; since then, the movement has grown into the Lord's Resistance Army (LRA) Insurrection. This rebel group, led by Joseph Kony, waged war not only against the Uganda People's Defense Force, but also against civilians residing in the area. As a consequence, the majority of northern Uganda's population has directly experienced mass violence, and more than 1.8 million civilians have lived in squalid camps as internally displaced persons (IDPs) [2]. In 2006, the Ugandan government began negotiating potential peace accords with the LRA, and a permanent cease-fire agreement was signed between the two parties in 2008. Despite signs that the conflict may be coming to an end, the impacts of war remain.

Years of protracted war have not only exposed northern Uganda's population to violence and displacement, but also have caused the collapse of economic and social structures, and disrupted the health care delivery system. Civilians living in IDP camps for years were dependent on relief aid, debilitated by malnutrition and preventable diseases [3]. Women and girls who were exposed to high rates of sexual violence, as well as a smaller proportion of men who were raped, suffer profound psychological and medical consequences [4]. Thousands of children were abducted, and many were forced to commit atrocities against their families and friends [5]. Prior studies suggest that perpetration of atrocities and experiences of rape are among the most psychologically destructive forms of violence suffered by children in war [6].

## Mental Health Needs and Services

War experiences and social disruption have had drastic effects on the psychological and social well-being of Ugandan civilians, placing many at considerable risk for persistent mental symptoms and psychiatric syndromes. Multiple psychiatric problems can be triggered by trauma and conflict [7]; however, most studies have focused on investigating post-traumatic stress disorder (PTSD) in northern Uganda and other post-conflict settings. Some researchers argue that focusing on a single disease entity diverts attention from social interventions that would help the majority of survivors adapt to the adverse conditions in post-conflict settings [8].

Studies conducted in northern Uganda have revealed a high burden of psychiatric symptoms in the general population. Recent population surveys in which individuals are evaluated with screening instruments estimate the prevalence of PTSD symptoms at 54%–75% and depressive symptoms at 44.5%–67% [9],[10]. Studies that clinically evaluate individuals with diagnostic interviews report lower rates of clinical syndromes of depression and PTSD. For example, Okello et al., using the Mini-International Neuropsychiatric Interview, compared formerly abducted adolescents to non-abducted controls and found that the abducted group had a greater prevalence of mental disorders including PTSD (26.8% versus 12.7%), major depression (19.5% versus 4.2%), and generalized anxiety disorder (13.4% versus 4.2%) [11].

It is important that prior to any type of intervention, psychiatric symptoms are differentiated from full blown psychiatric disorders. The former may be related to daily stressors such as poverty and structural adversity and thus may be amenable to psychological interventions [12], while the latter could be disaster induced or preexisting mental disorders, including mood disorders, substance use disorders, and acute and chronic psychoses that need specialized interventions. Indeed, there is widespread agreement that severely or chronically mentally ill people are often invisible in conflict and post-conflict settings, as well as being at very high risk [13].

In the Ugandan context, two studies have documented that a locally adapted western therapy had efficacy in treating PTSD and depressive symptoms among war-affected individuals [14],[15]. Moreover, culturally appropriate mental health assessment instruments have been developed and validated to screen for psychosocial needs in northern Uganda [16]. Yet, northern Uganda remains a region with massive unmet mental health needs and limited data on the impact of specialized interventions for severe mental disorders.

## The Potential Role of Public–Private Partnerships

Given that today's complex health challenges in resource-limited settings cannot be solved by governments alone [17], public–private partnerships (PPPs) are emerging as a strategy for leveraging the strengths of multiple sources in order to address health issues in low- and middle-income countries (LMICs) [18]. Non-governmental institutions throughout the world have long provided public assistance, either alongside government agencies or independently [18]. Increasingly though, private foundations, individuals, non- governmental organizations, and academic institutions have become involved in social and humanitarian efforts on a large scale [18].

PPPs may differ in their scope of activities, nature of partners, and objectives [19]. Some partnerships are between large multinational companies and international agencies involving broad strategies while others are more modest, occurring at the local level. Private partners can include private for-profit companies and individuals, private not-for profit organizations, donor organizations, and private donors, while public partners include governments at the national and local level, public organizations such as academic institutions, and community groups [20]. In global health delivery, many are formed with disease-specific objectives such as strengthening HIV/AIDS health services [21].

Unfortunately, PPPs to strengthen mental health services have not been well described in LMICs. In spite of the growing global burden of mental disorders, poor countries, particularly those in the African region, are ill-equipped to address mental health needs [22]. The extreme limitation of resources expended on mental health in war-affected LMICs is made more troubling by evidence that depression has an enormous impact on both morbidity and mortality and is the leading cause of chronic disease burden in LMICs [23].

## The Partnership

The Peter C. Alderman Foundation (PCAF) is a 501(c) (3) corporation dedicated to the memory of Peter C. Alderman, who was murdered by terrorists on September 11, 2001. He was 25 years of age when he died. Headquartered in the United States, its mission is to heal the emotional wounds of victims of terrorism and mass violence in post-conflict countries (http://www.petercaldermanfoundation.org/).

During the past decade, working in public–private partnership with ministries of health, the PCAF has trained and supervised physicians and other health care professionals in LMICs. Presently, in concert with its partners, the foundation operates eight psycho-trauma clinics in Cambodia, Uganda, Liberia, and Kenya. To date, PCAF has trained about 1,000 mental health professionals from 22 countries. Moreover, PCAF clinics and PCAF-trained personnel have treated over 100,000 victims suffering from the psychic wounds of war.

In 2005, the PCAF (the private partner) and the Ugandan government institutions (the public partner) initiated a collaboration seeking to deliver sustainable treatments for depression, PTSD, and other mental and neurological disorders in post-conflict, underserved areas. Contributions by each partner are summarized in Box 1.

**Box 1. Contributions made by each stakeholder in the public–private partnership**

**The Makerere University College of Health Sciences, Department of Psychiatry**

The department of psychiatry has made a number of contributions to the PPP. First, the department provides specialized practitioners including psychiatrists, psychologists, and social workers who provide the PCAF clinic staff with training in diagnosis and treatment of mental, neurological, and substance use (MNS) disorders and co-morbid medical problems in post-conflict settings. Second, these specialists have provided training in documentation of medical records, clinical monitoring and evaluation of treatment outcomes, and use of electronic patient data management systems. Third, these specialists have conducted cross-cultural adaptation of all screening tools used in each PCAF clinic. Finally, they have developed training curricula and provided continuing staff supervision.

**The Ministry of Health/Butabika National Mental Referral Hospital**

The Ministry of health not only ensures effective supervision, coordination, and monitoring of the Foundation's activities, but also links the Foundation with local governments, private and government hospitals, health training and religious institutions, and other stakeholders. The Butabika National Mental Referral Hospital provides specialists who train, support, and supervise PCAF clinic staff. Further, the hospital provides training space and psychotropic drugs and ensures transparency in hiring practices.

**The Peter C. Alderman Foundation**

***Training Programs.*** Through its annual Pan-African Conference on Psychotrauma, PCAF supports the training of doctors and other mental health professionals. These healers have gone on to train community lay workers and members of the village health teams who support them in their outreach activities. Training programs include basic training in the Harvard Program in Refugee Trauma 11-Point Toolkit in Healing Mass Violence, continuing professional education, and caregiver support.

***Employment Opportunities and Capacity Building.*** PCAF recruits and provides salary support to mental health workers including psychiatric nurses, psychiatric clinical officers, social workers, and trauma counselors who previously could not be absorbed into the government service, thereby boosting the numbers of mental health workers providing holistic primary mental health care in northern Uganda.

***Program Design.*** Working with its partners, PCAF provides leadership and strategic direction, liaises with district hospital administration officials, and provides ongoing quality assurance of the program.

**Civil Society: Religious Institutions, Faith-Based Organizations, and Non-governmental Organizations**

In some areas, contributions from civil society have included outpatient clinic space, technical support, outreach to towns and settlements, and in-patient beds. Pastoral care staffs have provided for group or individual spiritual healing sessions as needed.

## Methods

### Ethics Statement

PCAF trauma clinics were mainly created to offer a service, but not for research purposes. However, in the course of offering routine mental health care, we realized that an evaluation component would be essential to ascertain whether the services offered benefit the intended recipients. A research proposal was submitted to and approved by both the Makerere University College of Health Sciences research ethics committee and the Uganda National Council of Science and Technology (UNSCT). Given that it was impracticable to obtain informed consent for all service users receiving routine mental health care, that the research posed minimal risk, that the rights or interests of the patients would not be violated, and that their privacy and confidentiality or anonymity would be assured, both institutions waived the requirement of a signed consent form.

### Clinical Operations

Over a 6-year period (2005–2011), the partners established five psycho-trauma centers in the districts of Tororo (2006–2011), Gulu (2008), Kitgum (2009), Arua (2010), and Soroti (2011). Based at district hospitals, each PCAF trauma clinic employs a supervising psychiatrist, a psychiatric clinical officer, a psychiatric nurse, a social worker, and a trauma counselor/psychologist, all of whom received initial training at the Butabika National Referral Hospital. Furthermore, each clinic runs two outreach centers located within a radius of 50–80 km.

The psychiatric clinical officer performs a full clinical assessment including a structured psychiatric interview based on DSM-IV criteria, mental state examination, and a general medical examination. Depending on need, social and psychological assessments are made by the social worker and trauma counselor, respectively. The PCAF team reviews the entire evaluation and determines therapy. Treatments include psychopharmacology and psychotherapy as shown in Table 1.

**Table 1:** Demographic and clinical profiles of the Peter C. Alderman program participants by gender (N = 2,868).

Characteristics	Adults (18 years & over)			Children/Adolescents (0–18 years)			Total
	Male N = 940 n(%)	Female N = 1,050 n(%)	?^2^	Male N = 470 n(%)	Female N = 408 n(%)	?^2^	N = 2,868 n(%)
**Age (mean) (age range)**	34.5(19–79)	37.3(19–82)		13.4(5–18)	13.6(5–18)		
**Marital status:**							
Married	561(59.68)	518(49.33)	21.4*	43(9.15)	28(6.86)	1.5	1150(40.09)
Widowed	44(4.68)	260(24.76)	24.5*	15(3.19)	2(0.49)	0.1	321(11.19)
Divorced/separated	91(9.68)	182(17.33)	154.1*	15(3.19)	14(3.43)	8.4	302(10.53)
**Attending school**	230(24.47)	145(13.81)	36.8*	350(74.47)	293(71.81)	0.8	1018(35.49)
**Employment status:**							
Employed	517(55)	559(53.24)		76(16.17)	90(22.06)		1242(43.31)
Not employed	423(45)	491(46.76)	61.8*	394(83.83)	318(77.94)	5.1	1626(56.69)
**Vulnerable groups**							
Formerly abducted	306(32.55)	334(31.81)	0.1	72(15.32)	47(11.52)	2.7	759(26.46)
Victim of domestic violence	233(24.79)	465(44.29)	82.8*	109(23.19)	95(23.28)	0.1	902(31.45)
Suffered sexual violence	83(8.83)	247(23.52)	77.4*	32(6.81)	53(12.99)	9.5*	415(14.47)
Living in an IDP camp	510(54.26)	673(64.1)	19.9*	291(61.91)	244(59.8)	0.4	1718(59.9)
Former combatants	190(20.21)	70(6.67)	80.1*	37(7.87)	23(5.64)	1.7	320(11.16)
Orphans (N = 878)	-	-	-	158(33.62)	160(39.22)	2.9	318(36.22)
HIV positive	102(10.85)	160(15.24)	11.3*	9(1.91)	14(3.43)	2.6	285(9.94)
**No. of traumatic events experienced**							
≤3	93(9.89)	114(10.86)		29(6.17)	30(7.35)		226(9.30)
4–6	101(10.74)	177(16.86)		17(3.62)	30(7.35)		325(11.33)
>6	746(79.36)	759(72.29)	16.9*	424(90.21)	348(85.29)	6.8*	2277(79.4)
**Clinical diagnoses**							
Depression	369(39.26)	684(65.14)	133.4*	119(25.32)	125(30.64)	3.1	1297(45.22)
Epilepsy	284(30.21)	205(19.52)	30.5*	381(81.06)	285(69.85)	14.9*	1155(40.27)
PTSD	402(42.77)	538(51.24)	14.2*	62(13.19)	74(18.14)	4.1*	1076(37.52)
Alcohol/substance use disorders	200(21.3)	43(4.1)	135.8*	11(2.3)	1(0.3)	7.1*	256(9.0)
Non-lethal suicidal behavior	49(5.2)	71(6.8)	2.1	10(2.1)	19(4.7)	4.4*	149(5.2)
Bipolar disorder	70(7.5)	61(5.8)	2.2	8(1.7)	11(2.7)	1.1	150(5.2)
Schizophrenia	56(6.0)	32(3.1)	9.9*	5(1.1)	1(0.3)	2.2	89(3.1)
Other psychotic disorders§^a^	123(13.1)	92(8.8)	9.6*	20(4.3)	15(3.7)	0.2	236(8.2)
Other mental disorders^b^	103(11)	156(14.8)	6.7*	31(6.6)	42(10.3)	3.9*	332(11.6)
Childhood developmental disorders	-	-	-	55(11.7)	41(10.1)	0.6	159(5.5)
**Psycho-therapeutic interventions**							
Group counseling	66(7.0)	123(11.7)	12.7*	18(3.8)	16(3.9)	0.0	223(7.8)
Individual counseling	693(73.7)	915(87.4)	57.5*	311(66.2)	289(70.8)	2.2	2,208(77.0)
Family counseling	207(22.0)	232(22.1)	0.0	201(42.8)	141(34.6)	6.2*	781(27.2)
Traditional/faith healing	216(23.0)	230(22.0)	0.3	61(12.9)	45(11.0)	0.8	552 (19.3)
Home visits	34(3.62)	63(6.0)	6.1*	21(4.5)	16(3.9)	0.2	134 (4.7)
**Psychotropic medications**							
**Anti-psychotics**							
Haloperidol	167(18.0)	124(12.0)	56.2*	33(7.1)	31(7.6)	0.1	357(12.5)
Chlorpromazine	147(15.7)	57(5.6)	13.6*	22(4.7)	13(3.2)	1.8	240(8.4)
**Antidepressants**							
Amitriptyline	388(41.4)	658(63.2)	96.6*	65(14)	70(17.7)	4.2	1183(41.5)
Imipramine	100(10.7)	173(16.7)	14.8*	31(6.6)	37(9.1)	1.8	341(12.0)
Fluoxetine	19(2.0)	25(2.4)	0.32	3(0.6)	2(0.5)	0.1	49(1.7)
**Benzodiazepines**	102(11.0)	59(5.7)	19.0*	21(4.5)	17(4.2)	0.1	200(7.0)
**Anti-convulsants**	287(33.5)	206(22.7)	29.0*	365(79.7)	280(71.8)	7.7*	1143(43.6)

χ^2^: chi-square.

* Chi-square *p*-value≤0.05.

a Includes organic psychosis, paranoid psychosis, post-partum psychosis.

b Includes somatoform disorders, insomnia, bereavement, and other anxiety disorders such as generalized anxiety, panic anxiety.

Monthly counseling sessions are provided individually or in group. The sessions focus mainly on problem-solving, psycho-education, brief interventions for alcohol-use problems, and grief counseling. Group counseling consists of five monthly sessions. In the first session participants share trauma stories, and in the next two sessions they discuss positive and negative coping skills. In the fourth session the group receives psycho-education on the common mental, neurological, and substance use (MNS) disorders and their complications. Due to the high prevalence of HIV/AIDS in this population, pretest and post-test voluntary counseling and testing for HIV/AIDS is done. In the last session participants share stories of beneficial outcomes resulting from group counseling. Further, we take note of whether a patient is receiving spiritual healing sessions from either a traditional healer or faith healer.

### Clinic Monitoring and Evaluation System

Between August and December 2011, a cohort of 631 adult men and women with a history of war-related traumatic experiences was enrolled into four PCAF trauma clinics situated in four districts (Arua, Kitgum, Gulu, and Soroti) in northern Uganda. These four districts are comprised of different ethnic populations (Alur in Arua; Itesots in Soroti; and Luo in Gulu and Kitgum). They form part of a post-conflict northern region of Uganda that has endured more than two decades of brutal civil wars.

All individuals were requested to return for follow-up assessments at 3 and 6 months following initiation of care in the PCAF clinics. Two hundred thirty-eight were lost to follow-up (not returning for either follow-up assessment). These individuals were excluded from the present longitudinal analyses, which included data through the third visit (6-month assessment). Thus, the sample size for our final multivariate longitudinal models (including baseline through the third visit) was 375. Each individual could contribute up to three observations; the average number of observations was 2.4. The overall retention rate was 50% through the third visit.

### Covariates

A standardized structured questionnaire administered in the local language of each clinic population was used to collect data on a number of covariates in one-on-one, face-to-face interviews.

#### Socio-demographic variables

Socio-demographic variables were assessed using a standardized demographic questionnaire. The questionnaire asked about descriptive information including age, gender, and employment status. Employment status was categorized into “unemployed versus employed”. Marital status was categorized into “single versus married versus previously married (divorced, separated, or widowed).

#### Vulnerability groups

Among various vulnerabilities, we examined whether an individual was formerly abducted; was a former combatant; experienced sexual violence; experienced domestic violence; lived in an IDP camp.

#### Trauma events

War-related traumatic experiences were assessed using a locally developed 16-item trauma event checklist. Participants were asked whether they had experienced a given traumatic event or not. The trauma event checklist included items such as, “Has the patient been forced to torture others? Has the patient witnessed torture/killing of another person? Has the patient been forced to kill?” A variable indicating the number of traumatic events experienced by an individual was created and categorized as “less than three trauma events versus 3–6 trauma events versus more than 6 trauma events”.

#### HIV serostatus

Individuals were asked if they were aware of their HIV status. For those patients whose HIV serostatus was not known, pretest counseling was given before HIV serology was done.

#### Clinical variables

Clinical diagnoses and pharmacological and psychotherapeutic interventions were recorded in a clinical data form for each individual enrolled in a PCAF clinic. Variables indicating presence or absence of a given clinical diagnosis, psychotropic medication, and psychotherapeutic intervention were created.

#### Social worker home visits and use of traditional or faith healers

We asked individuals enrolled in PCAF trauma clinics to report whether they were concurrently receiving spiritual healing sessions from either a traditional healer or faith healer (Yes or No). Additionally, we noted whether individuals received social worker home visits (Yes or No).

### Outcome Variables

#### Depression symptoms

Depression symptoms were assessed using the self-reporting questionnaire (SRQ-20). The SRQ-20 has been successfully translated into at least 20 languages in several developing countries, with acceptable measures of reliability and validity [24]. The SRQ-20 has been adapted and validated among individuals enrolled in the PCAF clinic. In this study population, the measure attained a reliability coefficient (Cronbach α) of 0.97.

#### Post-traumatic stress symptoms

Post-traumatic stress symptoms were assessed using the locally adapted Harvard Trauma Questionnaire (HTQ). It has been successfully translated into several languages, with acceptable measures of reliability and validity [25]. In this study population, the measure attained a reliability coefficient (Cronbach α) of 0.95.

#### Functioning level

Functioning levels of individuals enrolled in PCAF clinics were assessed using a locally developed function assessment instrument [26]. Items were derived from qualitative interviews with individuals and their caregivers who were attending PCAF trauma clinics about their expectations regarding function outcomes. A five-item function assessment tool was piloted and field tested among 514 individuals randomly recruited from four PCAF clinics. The overall Cronbach α across the four clinic populations (Gulu, Kitgum, Soroti, and Arua) was 0.71. Work functioning and social functioning were the two factors of the assessment tool that were identified by principal component analysis. In each domain the respondent indicates their ability to perform a given task. Answers are coded on a three-point scale in which the responses were “No, I am not able” (0), “Yes, but not like before” (1), and “Yes, I am able to….” (2).

### Statistical Analyses

Patient data was entered into an EPIDATA database and analyzed using STATA statistical software (version 10; Stata Corp, College Station, TX). First, we conducted bivariate analyses using simple logistic regression models to obtain gender differences in baseline demographic, clinical, and treatment variables for 2,868 adults and children (Table 1).

Second, we used multivariate longitudinal regression models to examine changes in depression, post-traumatic stress, and functional outcomes measured across three time periods (baseline, 3-month, and 6-month) while adjusting for covariates associated with these outcomes. In these models, we used the generalized estimating equation analysis to account for correlated repeated measures within subjects, assuming a binomial distribution for the dichotomous outcomes of elevated depressive and post-traumatic stress symptoms and function scores. Three separate models were analyzed in which the dependent variables were change in the proportion of patients who have high depression scores (SRQ scores>6) and high PTSD scores (HTQ scores≥30) with a concomitant increase in the proportion of patients with high function scores (≥8) (Table 2).

**Table 2:** Longitudinal analysis of depression symptom scores, post-traumatic stress symptom scores and functioning scores over time (N = 375).

Characteristics	Depression Symptom Scores		Post-Traumatic Stress Symptom Scores		Functioning Scores	
	β Coefficient	SE	β Coefficient	SE	β Coefficient	SE
Intercept	8.80	0.66‡	26.22	1.52‡	0.83	0.39‡
Group counseling	1.61	0.84‡	1.43	1.48	1.25	0.39‡
Age>30 years	−0.02	0.45	0.2	0.99	−0.45	0.28
Previously Married	1.91	0.63‡	1.81	1.32	−0.41	0.39
Formerly abducted	0.66	0.39†	2.90	0.85‡	−0.12	0.24
Suffered sexual violence	0.54	0.45	3.75	1.01‡	−0.28	0.28
Living in an IDP camp	0.78	0.41‡	1.01	1.00	−0.30	0.34
HIV positive	0.51	0.43	−1.41	0.94	−0.25	0.31
Baseline functioning scores≥8	−1.21	0.38‡	−3.01	0.89‡	-	-
Received social worker home visits	0.35	0.51	1.44	1.38	0.40	0.34
**Slope terms**						
Overall change in outcome in 3 months	−3.78	0.28‡	−6.68	0.55‡	1.46	0.19‡
Overall change in outcome in 6 months	−6.45	0.38‡	−9.52	0.73‡	2.88	0.52‡

‡ *p*-value≤0.05.

† 0.05<*p*-value<0.10.

In each model, there is a dependent variable (a given clinical outcome e.g., depression scores, PTSD scores, or functioning scores), independent variables (baseline characteristics), and time (ordinal variable representing the change in the dependent variable for each additional unit of time [i.e., 3 months] over the three periods, and which is attributed to mental health interventions offered in the PCAF program). The baseline characteristics that were added to the model included patient baseline demographic, clinical, and treatment characteristics.

Given that the standard tests indicated that attrition for the three separate models was non-random, we proceeded to calculate inverse probability weights for each model. All multivariate longitudinal regression models included attrition weights to account for attrition bias.

## Results

Over a 6-year period PCAF program participants increased from 300 in 2007 to over 3,000 by June 2012; however, we have complete data for 2,868 individuals. Details of their socio-demographic, traumatic experiences, clinical diagnoses, and applied treatments disaggregated by age and gender are shown in Table 1.

Among adults, several gender differences have been observed. Women were more likely to be single (divorced, widowed, or separated) and unemployed and to reside in IDP camps. They were significantly more likely to experience domestic violence, sexual trauma, and higher rates of HIV infection.

Men were significantly more likely to have been abducted and used as combatants, to have witnessed the torture or killing of a relative, been deprived of food, water and medicine, have suffered greater loss and destruction of property, and to have been forced to perform rituals and torture others.

The most common diagnoses in PCAF trauma clinics were depression, PTSD, epilepsy, and alcohol and substance use. Women were significantly more likely to have depression, PTSD, and/or grief reaction, and men were more likely to suffer from alcohol and substance use disorders and/or major mental disorders (bipolar disorder and psychosis). Other mental disturbances were evenly divided between genders. Regarding therapy, men were more likely to receive antipsychotics, while women more likely to receive antidepressants and commonly opted for group counseling. Other forms of psychotherapy were equally divided between the genders.

**Figure 1:**
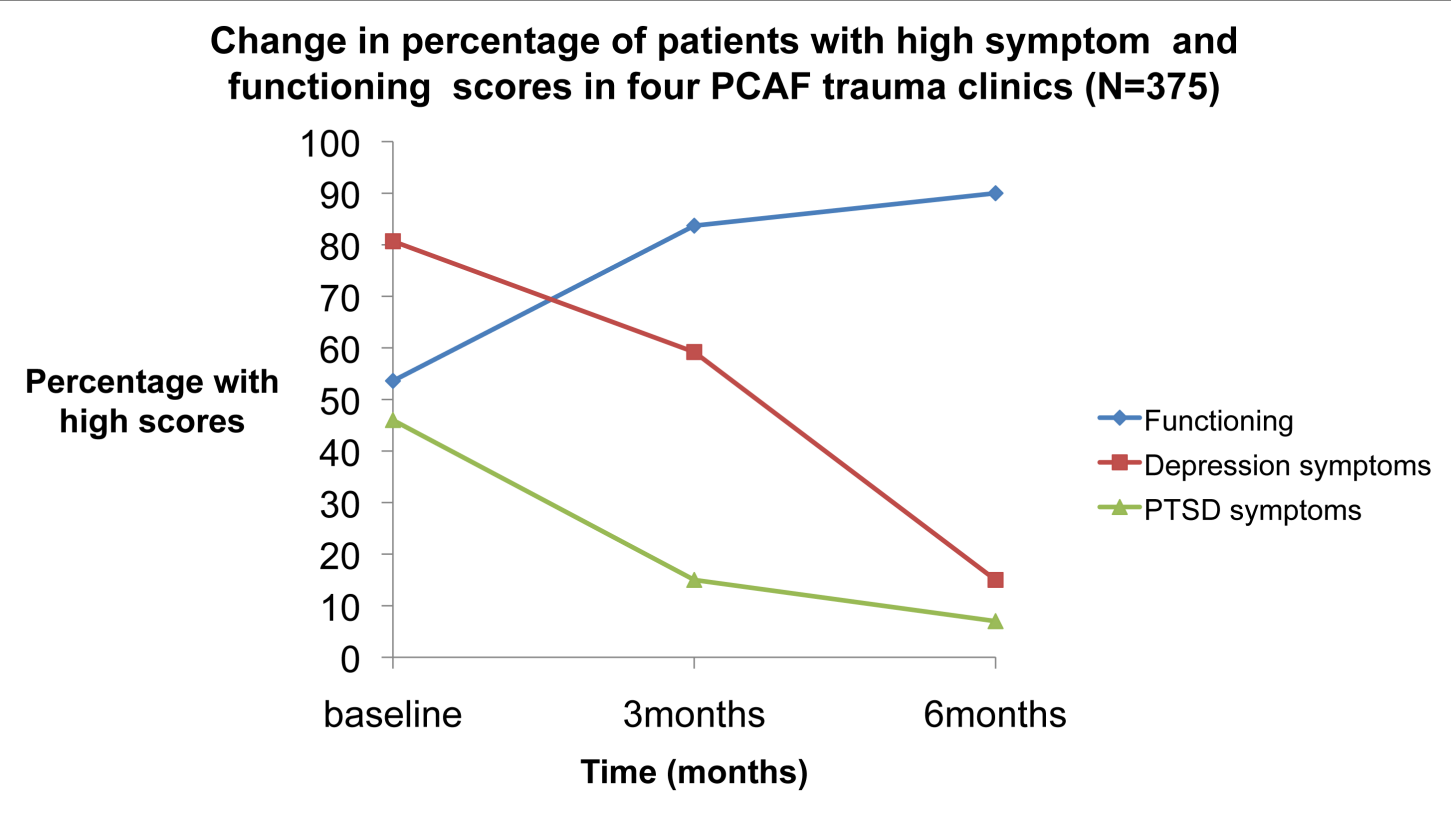
Percentage of individuals with high depression scores (SRQ scores>6), high PTSD scores (HTQ scores≥30), and high function scores (≥8) assessed at baseline, 3 months, and 6 months.

Figure 1 and Table 2 illustrate the decrease in the proportion of patients who have high depression scores (SRQ scores>6) and high PTSD scores (HTQ scores≥30) with a concomitant increase in the proportion of patients with high function scores (≥8) over a 6-month period after adjusting for covariates that were significantly associated with these outcomes.

## Discussion

It has been said that the single most important contribution of PPPs is the creation of social value [21]. In our experience, the PPP we developed has substantially increased access to mental health services for traumatized individuals living in several northern rural districts of Uganda. Successful PPPs are also shown to have an impressive record of being able to mobilize a significant amount of financial resources [17], which also rings true with our experience. This PPP has worked for 6 years to enhance Uganda's Ministry of Health's efforts to integrate mental health into its primary health care system, enabling the government to offer holistic care to its war-affected population. Consistent with findings from PPPs involved in strengthening health service delivery in LMICs [20] and other local initiatives to improve the mental health care system in rural areas in Uganda [27]–[29], our experience affirms that engaging the Ministry of Health as a stakeholder greatly facilitates dialogue between various actors in the health delivery system, and results in more comprehensive scale-up.

Previous researchers have identified stimulating research and development as one of the specific contributions that PPPs make towards promoting global health [17]. Partnering with the Department of Psychiatry, Makerere University made it possible to work with local experts to develop a monitoring and evaluation system to track clinical outcomes, thus enabling the partnership to have a demonstrable impact on improving the mental health of PCAF program participants. Further, local experts have not only conducted cross-cultural adaptations of all screening questionnaires, but have also developed a five-item function assessment instrument within the context of the local population's characteristics, culture, and preferences.

Consistent with previous findings from post-conflict settings [11],[12], data from the five psycho-trauma centers indicate that depression and PTSD are the most common mental health problems among PCAF program participants. However, the data also indicate substantial prevalence rates of other mental, neurological, and substance use disorders, as well as HIV infection. These findings confirm the importance of integrated management of mental disorders [30] and integrating HIV care into mental health services in post-conflict settings [31].

Gender differences in demographics, clinical diagnoses, and traumatic experiences observed among service users have more or less been described in other studies of traumatized populations [32]. This finding points to a need for more research into the impact of gender, especially in specific vulnerable groups, on clinical and function outcomes. This may facilitate the development of culturally sensitive gender-specific interventions for the local populations.

Further, we observed that combining western evidence-based therapies with traditional, culturally specific structures for reconciliation and reintegration in northern Uganda (such as matu oput, a cleansing ritual for returning child soldiers) resulted in positive outcomes. Our experience agrees with that of others regarding the important role of local rituals in the process of healing [33].

While PPPs hold much promise, they are not without challenges [34]. Indeed, while partnerships can lead to increased availability of services, there is also the potential for poor quality of services. During the early years of our project implementation, there was a lack of commitment from some staff who were not only absent from duty but also resisted the monitoring and evaluation strategies that were introduced in the project, lack of accountability, inadequate supervision of staff, inadequacy of drug supplies, and high attrition rates, each of which affected the quality of the services delivered.

To address such challenges, all stakeholders have endeavored to maintain a dialogue in which the partnership's goals and objectives are continuously reviewed and clarified. Performance measures have been established to enhance effectiveness, transparency, trust, and productivity.

## Limitations

Our analyses are potentially affected by selection bias given the use of non-random samples and the fact that the PCAF staff collecting data also provided the treatments. Further, the absence of a control group makes it impossible to account for natural changes in the outcomes that may occur over time in the absence of interventions provided. To isolate the effect of the PCAF treatment model, we need to conduct a randomized controlled trial in which the PCAF treatment model is compared with either a waiting list control group or those receiving usual mental health care. Further, our evaluation of this project is limited to descriptive statistical analysis of numbers treated and their characteristics, and single group cohort analyses; it would have been useful to evaluate the broader population level coverage and scaling up of the project [35], which would enable us to report on the increase in coverage for the target population over the period of the project.

## Conclusion

The pressing, unmet mental health needs in the post-conflict regions of northern Uganda can be addressed through PPPs, with implications for programming in other post-conflict settings. Public health goals, and the strategies used to attain them, must be compatible with the cultural values of the recipient populations. We believe that this partnership provides a model for integrating mental health care into the primary care system in LMICs. Moreover, we believe it is replicable, and can be rolled out in other post-conflict countries facing similar public health problems.

## Abbreviations

HTQ: Harvard Trauma Questionnaire
IDP: internally displaced person
LMIC: low- and middle-income country
PCAF: Peter C. Alderman Foundation
PPP: public–private partnership
PTSD: post-traumatic stress disorder
SRQ-20: self-reporting questionnaire
